# Headache as a Sentinel Signal After Cranial Radiotherapy: A Symptom-Driven Approach to Pathophysiology and Management

**DOI:** 10.3390/neurolint18070132

**Published:** 2026-07-10

**Authors:** Silviu Lunguț, Suzana Turcu, Cristiana Glavce

**Affiliations:** Francisc I. Rainer Institute of Anthropology, Romanian Academy, 050474 Bucharest, Romania; silviulungut47@gmail.com (S.L.); glavcecristiana@yahoo.fr (C.G.)

**Keywords:** cranial radiotherapy, headache, cerebral edema, neuro-oncology, migraine

## Abstract

Headache is a frequent and clinically relevant symptom in patients undergoing cranial radiotherapy, most often reflecting treatment-induced structural and inflammatory changes such as cerebral edema or radiation-related brain injury. Differentiating secondary headache from primary disorders, particularly migraine, is essential for appropriate management. This review aims to examine the pathophysiological mechanisms underlying headache following cranial radiotherapy, evaluate current pharmacological and complementary treatment strategies and highlight key aspects of differential diagnosis with migraine. Unlike existing literature that focuses primarily on radiological findings of radiation injury, this review adopts a symptom-driven approach, reframing headache as a critical clinical gateway for the early detection of structural complications. A structured narrative review of the literature was conducted using PubMed/MEDLINE, Scopus and Google Scholar to identify studies published between 2020 and 2025, focusing on cerebral edema, radiation-related complications, therapeutic approaches and migraine. Relevant clinical trials, systematic reviews and guidelines were included. Cerebral edema consistently emerges as the main mechanism of acute and subacute post-radiotherapy headache, whereas late-onset symptoms are most often linked to radiation necrosis. Corticosteroids remain first-line therapy, while bevacizumab has demonstrated benefit in steroid-refractory cerebral edema and radiation necrosis through inhibition of vascular endothelial growth factor (VEGF), thereby reducing vascular permeability and attenuating peritumoral edema. Its use in the context of cranial radiotherapy requires careful consideration, as the safety of concomitant administration with radiation has not been formally established, and headache itself is among its recognized adverse effects. Evidence for complementary therapies, including *Boswellia serrata* and plant-based compounds, remains limited. Migraine constitutes a distinct neurovascular disorder requiring careful differentiation from secondary headache in oncological patients. The review emphasizes headache as a clinically relevant indicator of underlying structural complications rather than an isolated symptom. Post-radiotherapy headache should be interpreted as a manifestation of underlying structural pathology. Accurate etiological diagnosis and individualized management are essential. Further research is needed to refine treatment strategies and clarify the role of complementary therapies.

## 1. Introduction

Headache is a common symptom among patients undergoing cranial radiotherapy and has a significant impact on both quality of life and clinical outcomes. The existing literature indicates that this symptom is most frequently associated with treatment-induced inflammatory changes, particularly cerebral edema, or, in later stages, with radiation necrosis. These processes lead to increased intracranial pressure, which explains the occurrence of headache, often accompanied by nausea, vomiting and other neurological manifestations [1,2,3].

**Figure 1:**
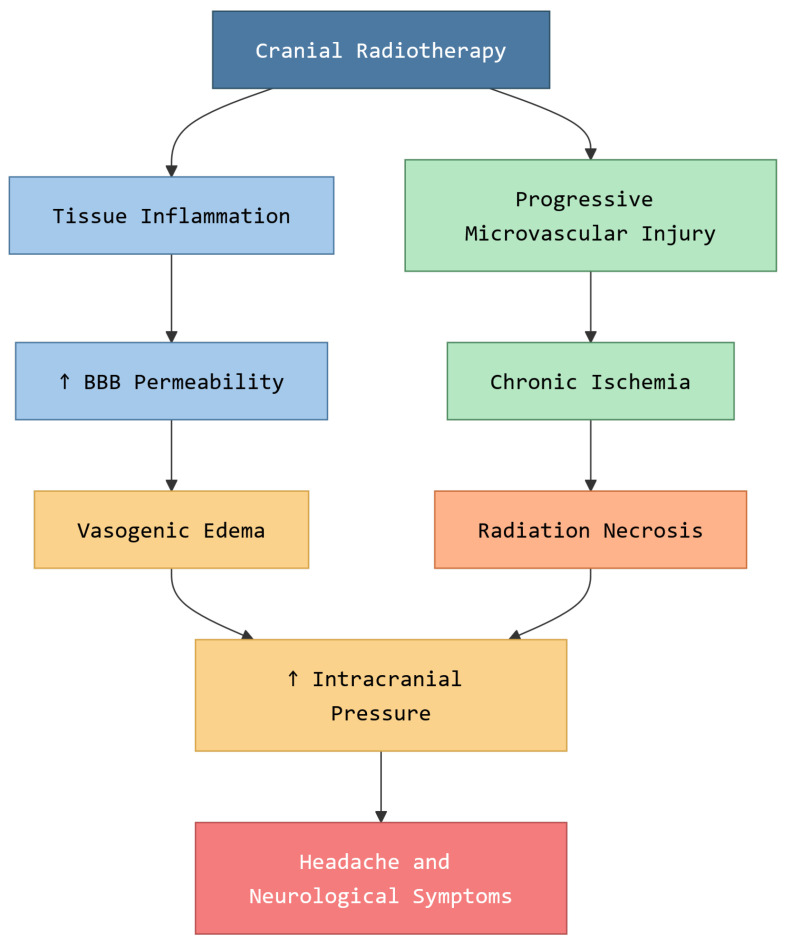
Pathophysiology of Post-Radiotherapy Headache.

Cranial radiotherapy induces complex changes in brain tissue, including inflammation, vascular injury and increased permeability of the blood–brain barrier. In the acute and subacute phases, these alterations contribute to the development of cerebral edema, which is considered the primary mechanism responsible for post-radiotherapy headache. In the late phase, occurring months to years after treatment, radiation necrosis may develop, characterized by tissue necrosis and microvascular damage, further contributing to persistent or progressive neurological symptoms [1]. Figure 1 illustrates the main pathophysiological pathways underlying post-radiotherapy headache.

The clinical significance of radiation-induced headache is increasing alongside improved patient survival rates. As systemic therapies evolve, patients live longer, allowing more time for late-phase complications like radiation necrosis to manifest, thus making long-term surveillance of new-onset headaches a clinical priority.

Between 2020 and 2025, the literature addressing headache in the context of cranial radiotherapy has focused predominantly on these underlying biological mechanisms rather than on headache as an isolated clinical entity. Recent studies emphasize that post-radiotherapy symptoms should be interpreted in relation to treatment-induced inflammatory and vascular changes and that optimal management depends on identifying the clinical and radiological cause of symptoms [2,4].

Recent recommendations from the European Association of Neuro-Oncology highlight that, in the presence of symptomatic cerebral edema, corticosteroids—particularly dexamethasone—remain the first-line treatment. These guidelines also stress the importance of using the lowest effective dose to minimize the adverse effects associated with prolonged corticosteroid therapy [2].

With regard to late complications, radiation necrosis is increasingly recognized as a major source of morbidity in patients treated with cranial radiotherapy, especially in the context of stereotactic techniques. Recent literature underscores the diagnostic challenges in distinguishing radiation necrosis from tumor progression, as well as the need for a multimodal approach in patient evaluation [4,5].

Regarding treatment strategies, the period between 2020 and 2025 has seen growing evidence supporting the role of bevacizumab in the management of radiation necrosis and steroid-refractory cerebral edema. Cranial radiotherapy induces local hypoxia and sustained neuroinflammation, both of which stimulate the upregulation of vascular endothelial growth factor (VEGF) in irradiated brain tissue. This radiation-induced VEGF overexpression increases endothelial permeability and promotes the accumulation of extracellular fluid, contributing directly to peritumoral and post-radiation edema. By binding to and neutralizing circulating VEGF, bevacizumab reduces vascular permeability and attenuates post-radiation edema through a mechanism driven by radiation-induced VEGF upregulation that is distinct from, though potentially overlapping with, its anti-tumoral anti-angiogenic effects. Systematic reviews and meta-analyses have demonstrated that this therapy can significantly reduce cerebral edema and improve neurological symptoms, including headache, in selected patients [6,7]. However, the safety of bevacizumab administered concomitantly with radiotherapy has not been formally established and headache is itself listed among its recognized adverse effects, an aspect that warrants careful clinical consideration when interpreting symptom evolution in treated patients. In parallel, recent research has explored complementary therapeutic approaches. A meta-narrative review published in 2025 suggests that *Boswellia serrata* may have beneficial effects on radiation-induced cerebral edema, although it also underscores the need for further clinical studies to confirm its efficacy and safety [8].

In this context, differentiating between radiotherapy-induced secondary headache and primary migraine becomes essential for appropriate clinical management. While the former is primarily driven by structural processes such as cerebral edema or radiation necrosis, migraine is a neurovascular disorder with distinct pathophysiological mechanisms and therapeutic strategies, including CGRP-targeting therapies [9,10].

From a clinical perspective, early recognition of headache following cranial radiotherapy is essential, as it may reflect underlying treatment-related complications such as cerebral edema or radiation necrosis. Misclassification of secondary headache as a primary disorder, including migraine, may lead to delays in appropriate diagnostic evaluation and initiation of targeted therapy. Such delays can result in progression of intracranial pathology, worsening neurological status and potentially avoidable morbidity. Timely differentiation between primary and secondary headache is therefore critical to guide appropriate imaging, optimize therapeutic decisions, and improve patient outcomes.

This review aims to analyze the pathophysiological mechanisms underlying headache following cranial radiotherapy, to evaluate current pharmacological and complementary treatment strategies, and to highlight the importance of differentiating this condition from migraine based on recent literature and clinical guidelines. Furthermore, this review specifically reframes headache not merely as a secondary symptom, but as a clinically relevant sentinel indicator of underlying radiation-induced structural pathology requiring prompt etiological evaluation.

While existing reviews typically address radiation necrosis or edema as primary endpoints, this paper distinguishes itself by positioning headache as the primary clinical entry point for diagnostic algorithms. We argue that headache serves as a ‘red flag’ or sentinel signal that precedes irreversible neurological damage, thus bridging the gap between subjective patient reporting and objective structural pathology.

## 2. Materials and Methods

### 2.1. Study Design

This study was conducted as a narrative literature review with structured search elements, aiming to synthesize current evidence on headache following cranial radiotherapy, including pathophysiological mechanisms, pharmacological management, complementary therapies and differential diagnosis with migraine. A narrative approach was chosen due to the heterogeneity of available studies and the inclusion of different types of evidence, such as clinical trials, systematic reviews and clinical guidelines.

### 2.2. Search Strategy

A literature search was performed using PubMed/MEDLINE, Scopus and Google Scholar to identify relevant publications. The PubMed/MEDLINE search combined Medical Subject Headings (MeSH) and free-text terms, including “cranial radiotherapy,” “brain irradiation,” “headache,” “cerebral edema,” “radiation necrosis,” “bevacizumab,” “dexamethasone,” “Boswellia serrata,” “acupuncture,” “migraine,” and “CGRP,” linked through Boolean operators (e.g., “cranial radiotherapy AND headache”, “radiation necrosis AND bevacizumab”). Filters were applied to limit results to human studies published in English between 2020 and 2025. The same combination of terms was adapted for Scopus and Google Scholar. Reference lists of relevant articles were manually screened to identify additional studies, including landmark publications published prior to this window and studies indexed under different terminology. Search terms were applied to all fields, not restricted to titles only. The relatively limited number of records retrieved (*N* = 75) reflects the combined effect of restrictive eligibility filters.

### 2.3. Inclusion and Exclusion Criteria

Studies were included if they met the following criteria:

1. Published between 2020 and 2025 (with the exception of landmark studies when necessary for context)
2. Written in English
3. Focused on cranial radiotherapy, cerebral edema, radiation necrosis, or headache in oncological patients
4. Addressed treatment approaches (pharmacological or complementary) or diagnostic considerations
5. Included clinical trials, systematic reviews, meta-analyses, or clinical guidelines

Exclusion criteria included:

- Case reports with limited generalizability
- Non-peer-reviewed sources
- Studies not directly related to central nervous system pathology or radiotherapy
- Articles lacking sufficient methodological clarity

### 2.4. Study Selection and Data Extraction

**Figure 2:**
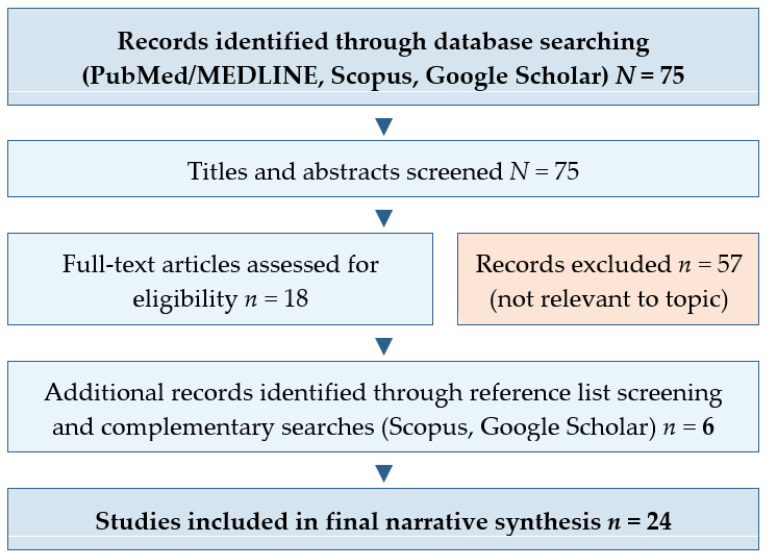
Flow diagram illustrating the study selection process.

Titles and abstracts were screened for relevance, followed by full-text review of selected articles. Studies were included based on their relevance to the objectives of the review. A combined PubMed/MEDLINE search using the terms described above yielded 75 unique records after automatic deduplication. Titles and abstracts of all records were screened against the predefined inclusion and exclusion criteria. Fifty-seven records were excluded because they were not directly relevant to the pathophysiology, treatment, or differential diagnosis of post-radiotherapy headache; this category included molecular and genetic studies on glioma biology without clinical correlation, as well as migraine-focused studies lacking any oncological context. The remaining 18 records were assessed in full text. Additional eligible studies and clinical guidelines, including landmark references not fully captured by the specific search terms used, were identified through manual screening of the reference lists of the selected articles and through complementary searches in Scopus and Google Scholar. A total of 24 studies were included in the final narrative synthesis (Figure 2).

Data extracted from the selected studies included:

- Study type (e.g., randomized controlled trial, systematic review, guideline)
- Population characteristics
- Main findings related to pathophysiology, treatment, or diagnosis
- Reported clinical outcomes (e.g., symptom improvement, reduction in edema)

### 2.5. Quality Assessment

Although a formal risk-of-bias assessment tool was not systematically applied due to the narrative nature of this review, the methodological quality of included studies was critically appraised. Greater emphasis was placed on high-level evidence, including clinical guidelines, systematic reviews, and meta-analyses, while observational studies and retrospective analyses were interpreted with caution. Particular attention was given to study design, sample size, consistency of findings, and clinical relevance when synthesizing the evidence.

### 2.6. Methodological Considerations

Given the narrative design, this review is subject to inherent limitations, including potential selection bias and the absence of quantitative synthesis. The heterogeneity of included studies, particularly regarding patient populations, radiotherapy techniques, and outcome measures, limits direct comparison across studies. Additionally, the focus on recent literature (2020–2025) may have excluded earlier foundational studies, although landmark references were incorporated where necessary to provide clinical context.

### 2.7. Data Synthesis

Given the heterogeneity of study designs and outcomes, a qualitative synthesis approach was adopted. Findings were grouped into thematic categories, including:

1. Pathophysiological mechanisms (cerebral edema, radiation necrosis)
2. Pharmacological treatment (corticosteroids, bevacizumab)
3. Complementary therapies (Boswellia serrata, acupuncture, etc.)
4. Differential diagnosis (migraine vs. secondary headache)

The synthesis aimed to provide an integrated understanding of current evidence rather than a quantitative comparison of results.

## 3. Results

The literature published between 2020 and 2025 on headache following cranial radiotherapy is characterized by considerable heterogeneity in study design, patient populations and reported outcomes. Most of the available evidence consists of systematic reviews, meta-analyses, clinical guidelines and a limited number of clinical trials, reflecting the complexity of this clinical entity and the difficulty of isolating headache as an independent outcome variable.

Across the reviewed studies, headache was not consistently reported as a primary outcome variable, nor was a standardized instrument systematically applied for its assessment. In most publications, headache was documented as part of broader neurological symptom scales or clinical toxicity grading systems, such as the Common Terminology Criteria for Adverse Events [3]. Patient-reported outcomes and validated headache-specific tools, such as the Numerical Rating Scale (NRS) or the Brief Pain Inventory (BPI), were rarely employed in the oncological radiotherapy literature reviewed. This heterogeneity in symptom reporting reflects a recognized gap in the field and limits direct comparison of headache burden across studies.

### 3.1. Acute and Subacute Mechanisms

Across the reviewed studies, cerebral edema is consistently identified as the principal mechanism underlying headache in the acute and subacute phases following cranial radiotherapy. Treatment-induced inflammation, vascular injury and disruption of the blood–brain barrier contribute to the accumulation of extracellular fluid within the brain parenchyma, leading to increased intracranial pressure and the development of neurological symptoms, including headache [2]. The severity of symptoms appears to correlate with the extent of edema observed on neuroimaging, with more pronounced edema being associated with a higher burden of clinical manifestations. In the acute phase, we propose interpreting headache as a surrogate marker for neuro-inflammation and blood–brain barrier disruption, providing a clinical window into the patient’s inflammatory status.

### 3.2. Late Effects and Radiation Necrosis

**Figure 3:**
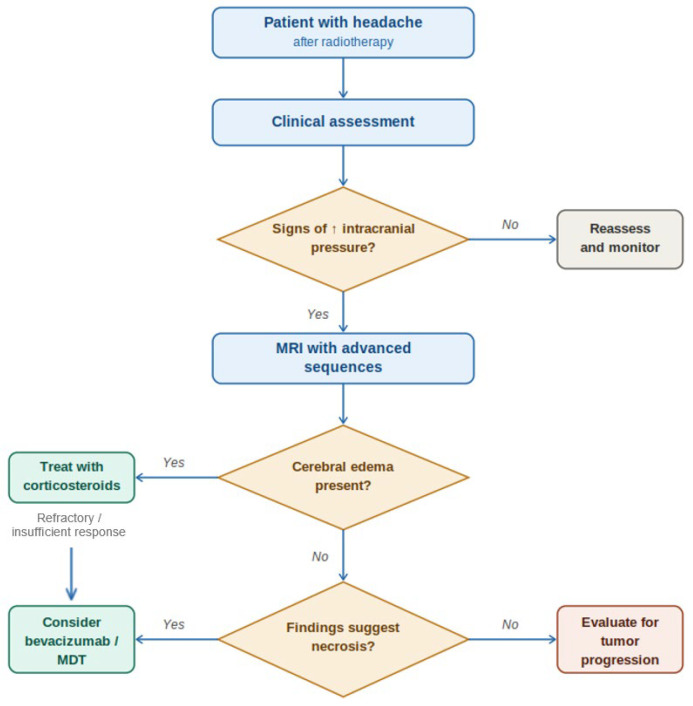
Simplified diagnostic algorithm for post-radiotherapy headache.

In contrast, in the late post-treatment phase, radiation necrosis emerges as a major contributor to persistent or progressive headache. The literature highlights the multifactorial pathogenesis of radiation necrosis, involving endothelial damage, ischemia and chronic inflammatory processes [4,5]. A recurring theme across studies is the diagnostic challenge posed by the overlap between radiation necrosis and tumor progression, as both conditions may present with similar clinical and radiological features. This overlap has led to the increased use of advanced imaging techniques and careful clinical correlation in order to achieve an accurate diagnosis. Figure 3 provides a simplified diagnostic algorithm for evaluating headache after cranial radiotherapy, a stepwise approach to assessing patients who develop headache following cranial radiotherapy.

In addition to the underlying biological mechanisms, dosimetric factors play a critical role in the development of post-radiotherapy complications. Dose–volume relationships have been consistently associated with the risk of both cerebral edema and radiation necrosis. In stereotactic radiosurgery (SRS), higher volumes of normal brain tissue receiving intermediate doses—specifically the V12 (volume of brain receiving ≥12 Gy)—have been correlated with an increased risk of injury [11,12]. Recent evidence suggests that a V12 exceeding 5–10 cm^3^ is a significant predictor of symptomatic radiation necrosis and associated clinical complications, including severe headache. Similarly, cumulative radiation dose and fractionation schemes influence the likelihood of tissue injury, with hypofractionated and high-dose regimens associated with a higher risk in susceptible patients.

The incidence and severity of radiation-induced complications also vary according to the radiotherapy technique employed. Compared with whole-brain radiotherapy (WBRT), focal techniques such as SRS and intensity-modulated radiotherapy (IMRT) allow for improved sparing of normal brain tissue but may still result in localized high-dose exposure associated with necrosis. Current recommendations from neuro-oncology and radiation oncology societies emphasize the importance of individualized treatment planning and careful dose–volume optimization to minimize toxicity while maintaining therapeutic efficacy [2,13]. These findings support cerebral edema as the dominant and most clinically actionable mechanism in early post-radiotherapy headache.

### 3.3. Pharmacological Treatment

In terms of treatment, corticosteroids—particularly dexamethasone—are consistently reported as the first-line therapeutic option for symptomatic cerebral edema. The reviewed literature confirms their effectiveness in reducing intracranial pressure and improving neurological symptoms, including headache [2]. However, concerns regarding the adverse effects associated with long-term corticosteroid use are frequently emphasized, leading to recommendations for the use of the lowest effective dose and gradual tapering when clinically feasible.

Bevacizumab has emerged as a major therapeutic option for patients with radiation necrosis or steroid-refractory cerebral edema. Systematic reviews and meta-analyses have shown consistent reductions in edema volume together with improvement in neurological symptoms and radiological findings following bevacizumab administration [6,7]. More recent multicenter and retrospective data also support meaningful clinical and radiological responses in symptomatic radionecrosis after stereotactic radiotherapy, including corticosteroid-unresponsive cases [14,15]. Despite these encouraging findings, variability in dosing regimens, treatment duration, and recurrence after discontinuation remains evident across studies, indicating the need for further standardization and prospective evaluation.

### 3.4. Complementary and Integrative Therapies

The role of complementary and alternative therapies remains less clearly defined. *Boswellia serrata* has emerged as a potential corticosteroid-sparing option because of its anti-inflammatory and antiangiogenic properties. Boswellia serrata’s therapeutic potential lies in its ability to inhibit the 5-lipoxygenase (5-LOX) enzyme, thereby reducing the production of pro-inflammatory leukotrienes that contribute to peritumoral and post-radiation edema [16]. In addition to narrative evidence synthesis, recent clinical data suggest that Boswellia may achieve meaningful radiographic responses in patients with radiation necrosis after stereotactic radiosurgery, with associated steroid-sparing potential in a subset of patients [8,17]. However, although these findings are encouraging, current evidence remains limited by the lack of randomized prospective trials, and important uncertainties persist regarding optimal formulation, dosing, treatment duration, and patient selection.

Similarly, acupuncture has been investigated as a supportive intervention in oncology, particularly for pain management. While recent meta-analyses demonstrate its effectiveness in migraine and chronic pain conditions, evidence specific to headache following cranial radiotherapy remains scarce [18]. Consequently, its role appears to be adjunctive rather than central in this clinical context.

### 3.5. Differential Diagnosis with Migraine

An important finding across the literature is the emphasis on differentiating secondary headache associated with cranial radiotherapy from primary headache disorders such as migraine. Migraine is characterized by episodic, often unilateral and pulsatile pain, frequently accompanied by nausea, photophobia and phonophobia and is mediated by neurovascular mechanisms involving CGRP pathways [9,10]. In contrast, headache related to radiotherapy is typically progressive, persistent and frequently associated with signs of increased intracranial pressure or focal neurological deficits [19].

In contrast to the limited number of validated options for the treatment of radiotherapy-induced secondary headache, the literature on migraine and other primary headache disorders has developed in recent years an expanded portfolio of adjunctive and complementary therapies, with varying levels of evidence. Recent meta-analyses on acupuncture have demonstrated a significant reduction in migraine attack frequency [18] and phytotherapeutic interventions have similarly shown benefit in primary headache disorders [20], suggesting an established role in the management of primary headache disorders. Furthermore, supplements such as magnesium and riboflavin are supported by international guidelines for migraine prevention, based on well-documented mitochondrial and neurovascular mechanisms [21].

These findings indicate that complementary therapies occupy an increasingly well-defined role in the management of primary headache disorders. While the underlying pathophysiological mechanisms of migraine differ fundamentally from those of radiotherapy-induced secondary headache, this contrast serves an important purpose within the present review: it highlights the risk of misclassifying secondary headache as a primary disorder and of applying treatment strategies that are not aligned with the structural and inflammatory etiology of post-radiotherapy symptoms. Understanding what works for migraine and why clarifies what remains unvalidated for oncological patients and underscores the need for dedicated research into complementary approaches for this population.

The reviewed literature indicates that headache following cranial radiotherapy should be understood primarily as a symptom of underlying structural and inflammatory processes rather than as an isolated clinical entity. The identification of the underlying cause—whether cerebral edema, radiation necrosis, or tumor progression—remains central to effective management.

## 4. Discussion

Clinically, the presence of new or progressive headache in patients undergoing cranial radiotherapy should prompt immediate evaluation for treatment-related complications rather than empirical symptomatic treatment. The available evidence indicates that headache following cranial radiotherapy should not be interpreted as an isolated symptom, but rather as a manifestation of underlying pathophysiological processes, most notably cerebral edema and radiation necrosis. The consistency with which these mechanisms are reported across recent literature reinforces the importance of adopting an etiological framework in both diagnosis and management [2,4].

Expanding this differential landscape, an essential but rare diagnostic consideration is the SMART syndrome (Stroke-like Migraine After Radiation Therapy) [22]. This entity typically manifests months to years after cranial radiotherapy and is characterized by migraine-like headaches, focal neurological deficits, seizures, and reversible cortical MRI signal changes, often mimicking stroke or tumor progression. The pathophysiology of SMART syndrome remains incompletely understood but is thought to involve cortical spreading depression triggered by radiation-induced cortical hyperexcitability and neurovascular dysfunction. Its clinical presentation, namely episodic, lateralized headache with transient neurological symptoms, may be misinterpreted as a primary migraine or as tumor recurrence, leading to diagnostic delay and inappropriate management [23]. SMART syndrome thus exemplifies the central argument of this review: that radiation-induced headache syndromes, even when superficially resembling primary headache disorders, reflect an underlying structural and neurovascular pathology that demands prompt etiological evaluation.

The distinct value of this review lies in its clinical-first perspective. In current oncological practice, headache is frequently dismissed as a non-specific side effect or mismanaged as a primary headache disorder. By synthesizing recent data from 2020 to 2025, we demonstrate that post-radiotherapy headache is rarely idiopathic and that its accurate interpretation as a symptomatic proxy for blood–brain barrier disruption and localized pressure changes has direct consequences for clinical decision-making.

In the acute and subacute phases, cerebral edema emerges as the dominant contributor to symptom development. The effectiveness of corticosteroids, particularly dexamethasone, in reducing edema and alleviating neurological symptoms is well established and continues to be supported by contemporary guidelines [2]. However, their use requires careful consideration given a well-documented adverse effect profile, including immunosuppression, hyperglycemia, proximal myopathy, neuropsychiatric symptoms, and increased susceptibility to infections. These concerns have led to growing interest in dose minimization strategies and steroid-sparing alternatives.

In the late phase, radiation necrosis represents a more complex and challenging condition. The reviewed studies highlight the diagnostic difficulty in distinguishing radiation necrosis from tumor progression, which carries significant implications for patient management. Although advanced imaging techniques such as perfusion MRI, MR spectroscopy, PET imaging, and MRI-based contrast clearance analysis can improve diagnostic confidence, no single modality provides definitive discrimination in all cases, and histopathology remains the gold standard in unresolved situations [4,5,24,25]. This diagnostic uncertainty has direct therapeutic consequences, as misclassification may lead either to delayed treatment of radionecrosis or to inappropriate escalation of oncological therapy.

Among steroid-sparing strategies, bevacizumab has shown the most consistent benefit. Its therapeutic rationale in the post-radiotherapy setting rests on the observation that cranial irradiation induces local hypoxia and sustained neuroinflammation, which in turn drive the upregulation of VEGF in irradiated brain tissue, leading to increased endothelial permeability and edema formation. By neutralizing circulating VEGF, bevacizumab reduces vascular permeability and attenuates edema formation, an effect that in the post-radiotherapy setting operates through radiation-induced VEGF upregulation rather than exclusively through inhibition of tumor angiogenesis, though the two mechanisms may coexist in clinical practice. Systematic reviews and meta-analyses have reported significant reductions in edema volume alongside meaningful clinical and radiological improvement in symptomatic radiation necrosis, including corticosteroid-refractory cases [6,7]. Surgical approaches such as laser interstitial thermal therapy (LITT) and resection may be considered when diagnostic uncertainty persists or when lesions prove refractory to medical management. LITT in particular has emerged as a minimally invasive option that allows both tissue diagnosis and targeted thermal ablation under imaging guidance [26,27,28,29,30]. Other adjunctive strategies, including hyperbaric oxygen therapy, have been proposed, but supporting evidence remains scarce and heterogeneous.

The available evidence for *Boswellia serrata* suggests potential anti-inflammatory effects through inhibition of leukotriene synthesis and modulation of inflammatory pathways, with a possible role in reducing radiation-induced cerebral edema [8]. Similarly, acupuncture has demonstrated efficacy in migraine and chronic pain conditions but lacks robust evidence in the specific context of post-radiotherapy headache [18]. The current level of evidence for both approaches remains limited by small sample sizes, heterogeneous study designs, and the absence of large-scale randomized controlled trials. Moreover, the use of complementary therapies in oncological patients requires careful evaluation given the potential for interactions with standard treatments.

Advances in migraine research, particularly the development of CGRP-targeting therapies including monoclonal antibodies and small-molecule antagonists, have significantly improved the management of primary headache disorders [9,10]. These therapeutic strategies are not directly applicable to secondary headaches resulting from radiotherapy-related complications. This distinction carries important clinical implications, as misclassification of post-radiotherapy headache as migraine may lead to inappropriate treatment and, critically, to delayed identification of potentially serious underlying structural pathology. Understanding the therapeutic landscape of migraine therefore serves not as a treatment template for oncological patients, but as a diagnostic contrast that clarifies the fundamental differences between primary neurovascular disorders and radiation-induced secondary headache syndromes, underscoring the need for dedicated research in this area.

The management of headache following cranial radiotherapy ultimately requires a multidisciplinary approach, integrating neuro-oncology, neurology, medical imaging, neurosurgery, and supportive care. Such collaboration allows for comprehensive patient evaluation, optimization of diagnostic strategies, and individualization of therapeutic decisions. Future research should prioritize the development of standardized protocols for headache assessment in oncological patients, prospective evaluation of both pharmacological and complementary therapies, and improved diagnostic tools for differentiating radiation necrosis from tumor progression. Establishing headache as a formal clinical endpoint in radiotherapy trials would represent a meaningful step toward bridging the gap between subjective symptom reporting and objective structural pathology.

### Limitations

This review has several limitations. First, the heterogeneity of the included studies limits the ability to perform direct comparisons or draw definitive conclusions. The reliance on narrative synthesis rather than quantitative meta-analysis reflects both the variability of available data and the scope of the review. Although the focus on recent literature (2020–2025) ensures relevance, it may have excluded earlier landmark studies that continue to inform clinical practice. Also, the reviewed studies did not consistently employ validated instruments for headache assessment, precluding any systematic evaluation of symptom severity or treatment response specifically related to headache as an outcome. This heterogeneity in symptom reporting represents a recognized gap in the field and underscores the need for well-designed prospective studies evaluating both pharmacological and complementary therapies, as well as for improved and standardized diagnostic tools to differentiate radiation necrosis from tumor progression.

## 5. Conclusions

Headache following cranial radiotherapy represents a complex clinical manifestation that reflects underlying structural and inflammatory processes rather than an isolated symptom. The evidence reviewed indicates that cerebral edema is the primary mechanism in the acute and subacute phases, while radiation necrosis plays a central role in late-onset and persistent symptomatology.

Current management strategies remain largely etiological. Corticosteroids continue to represent the first-line treatment for symptomatic cerebral edema, whereas bevacizumab has emerged as a valuable therapeutic option in cases of radiation necrosis or steroid-refractory edema. Despite these advances, important gaps remain regarding optimal treatment protocols and long-term outcomes.

Complementary and alternative therapies, including *Boswellia serrata* and plant-based compounds, show potential benefits but are currently supported by limited and heterogeneous evidence. Similarly, while acupuncture and other non-pharmacological approaches may contribute to symptom control, their role in post-radiotherapy headache remains adjunctive and insufficiently validated.

The differentiation between secondary headache related to cranial radiotherapy and primary headache disorders such as migraine is essential for appropriate clinical management. Advances in migraine research, particularly the development of CGRP-targeted therapies, highlight the distinct pathophysiological and therapeutic frameworks of these conditions and underscore the importance of accurate diagnosis.

Overall, effective management of headache in patients undergoing cranial radiotherapy requires a multidisciplinary and individualized approach, guided by clinical evaluation and imaging findings. The use of complementary therapies (non-pharmacological supplements, acupuncture) in oncological patients should be carefully evaluated due to potential interactions with standard treatments.

Through a ‘Sentinel Signal’ framework, this review reframes headache as the earliest detectable clinical warning of radiation-induced brain injury, often preceding irreversible neurological deficits. We argue that dismissing headache as a generic side effect or mismanaging it as a primary migraine leads to a critical diagnostic delay; such failure to recognize the underlying structural pathology results in the closure of the optimal therapeutic window for initiating life-saving interventions like corticosteroids or bevacizumab.

Future research should focus on the development of standardized treatment protocols, improved diagnostic tools for differentiating radiation necrosis from tumor progression and high-quality clinical trials evaluating both pharmacological and complementary therapies.

## Data Availability

No new data were created or analyzed in this study. Data sharing is not applicable to this article.
